# Fatigue in individuals with knee osteoarthritis: Its relationship with sleep quality, pain and depression

**DOI:** 10.12669/pjms.35.4.383

**Published:** 2019

**Authors:** Tulay Kars Fertelli, Fatma Ozkan Tuncay

**Affiliations:** 1 Dr. Tulay Kars Fertelli, Cumhuriyet University, Health Sciences Faculty, 58140 Sivas, Turkey; 2 Dr. Fatma Ozkan Tuncay, Cumhuriyet University, Health Sciences Faculty, 58140 Sivas, Turkey

**Keywords:** Knee Osteoarthritis, Fatigue, Sleep quality, Pain, Depression

## Abstract

**Objective:**

This descriptive cross-sectional study was carried out to investigate the relationship between fatigue, and sleep quality, pain and depression in patients with knee osteoarthritis.

**Methods:**

A cross-sectional, correlational design was used. The study sample comprised 151 individuals with knee osteoarthritis and 147 healthy individuals. To collect the study data, the Sociodemographic Characteristics Questionnaire (SCQ), Visual Analogue Scale to Evaluate Fatigue Severity (VAS-F), Pittsburgh Sleep Quality Index (PSQI), Visual Analogue Scale and Beck Depression Inventory (BDI) were used. To analyze the study data, appropriate tests were used.

**Results:**

It was found that the patients with knee osteoarthritis had higher fatigue scores, and higher PSQI and BDI total scores than did the healthy participants, and that the difference between them was significant (p<0.05). There was a positive correlation between fatigue score and PSQI, VAS and BDI scores (p<0.05).

**Conclusion:**

The participants with knee osteoarthritis suffered fatigue more than did the healthy participants. This fatigue suffered by them affected their sleep quality, pain and depression negatively.

## INTRODUCTION

Knee Osteoarthritis (KOA) is stated to be an important health problem for the aging population of the world.^1^ The knee OA decreases quality of life and leads to many problems such as disability and fatigue. Fatigue is a common complaint by people having rheumatic diseases and is an important indicator of quality of life.^2^,^3^ Approximately half (47%) of the individuals with osteoarthritis (OA) suffer fatigue.^4^,^5^ The 65 years and over people with OA have been found to suffer fatigue four times more than do healthy people.^5^,^6^ On the other hand, assessment of fatigue in the provision of OA care is often neglected.^2^ In the literature, it is stated that rather less attention has been paid to this issue and thus not much research has been devoted to the subject.^2^,^4^,^5^,^7^ Today, the review of studies shows that a small number of studies focus on individuals with OA receiving rheumatological care. Therefore, it is important to identify the factors affecting OA-related fatigue. Fatigue might be a primary outcome of OA, or it may be due to the side effects of drugs or sleeplessness.^2^

Sleeplessness is a risk factor for fatigue in OA.^5^,^8^ The problem of sleeplessness can occur in most of the rheumatic diseases, and its main cause is pain.^9^ Pain that increases with activity and decreases at rest in the early stage of the disease becomes more intense and resistant as the disease progresses. If pain still exists at rest, it can awaken the sufferer at night. Approximately 30% of individuals with OA are reported to experience night pain.^10^ Studies have shown that arthritis affects sleep quality adversely. These studies have also shown that patients with arthritis have sleeping problems such as difficulty in falling asleep, frequent sleep disruption and early morning awakening.^9^,^11^,^12^ The literature review indicates that although the majority of studies have been conducted on sleep quality in patients with rheumatoid arthritis, the number of studies investigating sleep quality in patients with KOA is low.^9^ It is pointed out that pain, joint stiffness, functional limitation, sleeplessness and fatigue can affect each other,^2^,^8^,^9^ which may have negative effects on the psychological wellbeing and that one of these negative effects is depression.^5^

Depression is seen in two-thirds of chronic rheumatologic patients.^13^ Depression risk is further increased in patients if OA is accompanied with chronic pain. When fatigue is studied in this respect, it is also important to determine the relationship between fatigue and depression. It is stated that there may be a many-sided interaction between clinical and psychological symptoms such as insomnia,^9^ fatigue^5^ and pain^3^ experienced by individuals with OA.^8^,^9^ Fatigue in OA has been reported to be related with physical and psychological factors, but what this relationship stems from is not clear, and studies relevant to this issue are very few^4^,^14^ However, in order to successfully manage fatigue, which has been shown to be the most distressing condition in the management of chronic diseases, it is important to determine these interactions.^2^,^4^ Thus, the present study was performed to identify the relationship between fatigue, and sleep quality, pain and depression in patients with KOA.

## METHODS

This study was conducted using descriptive, cross sectional and correlation research designs. The duration of study was nine months i.e. November 2017 till July 2018. The study population comprised outpatients with OA who were treated in the Physical Therapy and Rehabilitation, and Rheumatology Polyclinics of a university hospital. Of the patients in the study population, those diagnosed with KOA, aged 25 years and over, able to communicate, walk and move, agreeing to participate in the study, and not in an acute painful period were included in the study sample. The sample size was calculated in the Stat Direct program taking α=0.05, β=0.20 and 1-β=0.80 (p=.80112) and the study was carried out with 151 individuals.

To determine whether the participants with OA differed from the normal population, of the relatives of these participants who accompanied them, 147 healthy volunteer subjects with similar socio-demographic characteristics and no health problems were assigned to the control group using the non-probability sampling method. Thus, the study sample included 298 individuals.

To collect the study data, Sociodemographic Characteristics Questionnaire (SCQ) developed by the researcher, Visual Analogue Scale to Evaluate Fatigue Severity (VAS-F), Pittsburgh Sleep Quality Index (PSQI), Visual Analogue Scale and Beck Depression Inventory (BDI) were used.

### SCQ

This questionnaire, prepared by the researcher based on the literature, consists of 14 items questioning the patient’s sociodemographic characteristics such as age, and gender.

### VAS-F

The scale was developed by Lee et al.^15^ and adapted into Turkish by Yurtsever.^16^ The high score for the fatigue subscale and the low score for the energy subscale indicate that the severity of fatigue is high. Cronbach’s α internal consistency coefficient was 0.90 for the fatigue subscale and 0.74 for the energy subscale.^16^

### PSQI

The PSQI assesses the sleep quality within the last one month with 19 questions. It consists of seven components: subjective sleep quality, sleep latency, sleep duration, sleep efficiency, sleep disturbances, use of sleeping medication, and daytime dysfunction. The total score ranges from 0 to 21. Scores six or higher indicate impaired sleep quality. The scale was adapted into Turkish by Agargun.^17^

### VAS

The VAS is very commonly used to measure pain intensity ranging between 0 (no pain) and 10 (irresistible pain). While the VAS score of “0” indicates no pain, 1-4 indicates mild, 5-6 indicates moderate, and 7-10 indicates severe.^18^

### BDI

This 21-item inventory is a self-report questionnaire. The items are scored between zero and three according to the severity of the depression. The minimum and maximum possible scores to be obtained from the overall inventory are 0 and 63 respectively. While a score of 0–13 indicates minimal depression, 14–19 indicates mild depression, 20–28 indicates moderate depression, and 29–63 indicates severe depression. The scale was adapted into Turkish by Hisli.^19^

The patients with OA and healthy individuals after being informed about the study were administered SCQ, VAS-F, PSQI, VAS and BDI. The scales were administered by the researchers using interview techniques. The administration of the scales took an average of 30-35 minutes for each participant.

### Data Analyses

Data was analyzed using SPSS® 23. While the parameters for both groups were compared, the significance of the difference between two means and Chi-square test were used. To determine the relationship between fatigue, and sleep quality, pain and depression scores of the groups, the Pearson correlation test was used. P value of <0.05 was considered statistically significant.

### Ethical Consideration

The study was approved by the Cumhuriyet University’s Clinical Research Ethics.

## RESULTS

The mean age of the participants with OA was 53.93+6.84. Of them, 84.8% were female, 75.5% were housewives, 37.7% were primary school graduates. On the other hand, the mean age of the healthy participants was 52.85+7.95%. Of them, 91.8% were female, 93.2% were housewives and 31.3% were primary school graduates. According to the cut-off value of the total scores for the VAS-F, PSQI and BDI, of the participants with OA, 81.5% had high fatigue levels, 78.1% had high energy levels, 64.9% had deteriorated sleep quality (PSQI >5), 45% had moderate depression and 73.6% had severe pain. Of the healthy participants, 78.2% had low fatigue levels, 96.6% had high energy levels, 83.7% had no deterioration in sleep quality and 55.8% had mild depression.

Table-I compares the scores the participants with KOA obtained from the VAS-F, PSQI, VAS and BDI with those obtained by the healthy participants. The participants with KOA had higher levels of fatigue (80.60±23.89) and lower energy levels (32.35±10.30). The scores they obtained from the PSQI and its subscales except for the sleep medication subscale (6.77±3.02) and from the BDI (16.83±7.19) were higher, and the difference was significant (p<0.05). The participants with KOA also suffered severe pain (7.68 ±1.77).

**Table I:** Comparison of the mean scores the participants with knee osteoarthritis and the healthy participants.

Scales	Participants with knee osteoarthritis (n=151) Mean±SD	Healthy individuals (n=147) Mean±SD	t^a^	p

The Visual Analogue Scale to Evaluate Fatigue Severity				
Fatigue	80.60±23.89	39.23±14.77	17.91	0.000*
Energy	32.35±10.30	47.26±13.32	10.81	0.000*
Pittsburgh Sleep Quality Index	6.77±3.02	3.99±1.74	9.66	0.000*
Subjective sleep quality	1.29±0.73	0.69±0.50	8.23	0.000*
Sleep latency	1.42±0.90	0.42±0.62	11.06	0.000*
Sleep duration	1.18±0.87	0.72±0.64	5.12	0.000*
Habitual sleep efficiency	0.66±0.87	0.48±0.57	2.07	0.039*
Sleep disturbances	1.19±0.61	0.51±0.52	10.11	0.000*
Use of sleeping medication	0.29±0.68	0.18±0.38	1.78	0.081
Daytime dysfunction	0.70±0.78	0.94±0.69	2.75	0.006*
Beck Depression Inventory	16.83±7.19	15.24±5.72	2.10	0.036*
Visual Analogue Scale	7.68±1.77	-	-	-

* = p<0.05

The analysis of the mean scores obtained from the VAS-F, PSQI, VAS and BDI by the participants with KOA revealed a positive correlation between fatigue and the PSQI, VAS and BDI scores (p<0.05), and a negative correlation between energy and the PSQI, VAS and BDI scores (p<0.05). When the correlation coefficients were analyzed, a moderate relationship was found between fatigue and PSQI (r=0.685) and a weak relationship between fatigue and VAS (r=0.330) and BDI (r=0.440). There was a weak significant relationship between energy and fatigue and PSQI (r=0.685), VAS (r=0.330) and BDI (r=0.440) (Table-II).

**Table II:** Correlation between the mean VAS-F, PSQI, VAS, BDI scores obtained by the participants with knee osteoarthritis.

Evaluation scores	VAS-F		PSQI		VAS		BDI	

VAS-F	p	r	p	r	p	r	p	r
Fatigue	-	1	0.000	0.685*	0.000	0.330*	0.000	0.440*
Energy	0.000	-0.314*	0.000	-0.362*	0.000	-0.339	0.000	-0.380*

*Note*: The Visual Analogue Scale to Evaluate Fatigue Severity: VAS-F, Pittsburgh Sleep Quality Index: PSQI, Visual Analogue Scale: VAS, Beck Depression Inventory: BDI,

* = p<0.05.

## DISCUSSION

In the present study was found that individuals with KOA experience more fatigue, insomnia and depression than do healthy individuals. In several studies, it has been determined that individuals with OA experience fatigue,^9^,^10^,^13^ sleeping problems,^9^-^11^ and depression more 9,10,20. In a study investigating the relationship between fatigue, sleep, pain and depression in patients with rheumatoid arthritis and OA, the patients with OA were found to have more fatigue, sleep disturbance, pain and depression than the patients with rheumatoid arthritis.^13^

The fatigue is experienced by all people, but it is particularly prevalent in individuals with OA.^7^,^13^ In the present study, the participants with KOA obtained higher scores from the VAS-F and experienced fatigued more than did the participants in the healthy control group. In other studies, similar results were obtained.^10^,^20^ For instance, individuals with KOA experienced mild levels of fatigue in Sarıyıldız et al.’s study^9^ and moderate levels of fatigue in Murphy et al.’s study.^8^ However, in the present study, the participants with KOA experienced high levels of fatigue. The individuals in the aforementioned studies experienced fatigue; however, the level of fatigue varied from one study to another, which might be due to differences in the measurement tools used or due to their cultural characteristics. Another result obtained in the present study was that there was a positive relationship between fatigue and deterioration in sleep quality, pain and depression, and that there was a negative relationship between energy and deterioration in sleep quality, pain and depression. It has been determined that there is a relationship between knee pain and sleep disturbance in OA. Patients with OA have also been shown to exhibit problems related to falling asleep, sleep care and early morning awakening.^12^ Relevant studies have also shown that there is an important relationship between fatigue and sleep disturbances.^7^,^13^,^21^ Similar results obtained in this present study suggest that application of therapeutic approaches to sleep disturbances in OA management would be an appropriate intervention to control fatigue in these patients. In Asian culture, fatigue is considered as the natural result of aging, which may have affected these results.^22^

Another significant factor affecting fatigue is pain.^21^ In the present study, it was determined that fatigue affected pain and that there was a relationship between them. In their studies, Murphy et al.^14^ found similar results. In several other studies, it was also found that there was a relationship between pain and fatigue in patients with KOA.^21^,^24^ In their study conducted with 348 patients, Allen et al.^23^ used the VAS-fatigue and found a relationship between fatigue and pain. These results suggest that in the management of fatigue in individuals with KOA, pain is a factor that should be brought under control.

Fatigue occurs as a result of a complicated interaction of medical, physical, and psychiatric factors.^22^ Depression, one of the psychological factors, significantly affects the pain experienced by people with KOA. Those with high levels of depression have been determined to experience more pain. It is stated that pain can cause patients to experience more fatigue.^20^ In the present study, a significant relationship was determined between fatigue and depression scores of the participants with KOA. This result is consistent with those of other studies.^7^,^13^,^20^

## CONCLUSION

The results of the present study show that fatigue is a common symptom in individuals with OA and that this symptom leads to deterioration in sleep quality, pain and depression in individuals with OA.

### Author contributions

**TKF:** Conceived, designed, and performed statistical analysis.

**FOT:** Helped in analysis and interpretation of data, final approval of the manuscript.
